# Climate factors driven typhus group rickettsiosis incidence dynamics in Xishuangbanna Dai autonomous prefecture of Yunnan province in China, 2005–2017

**DOI:** 10.1186/s12940-019-0558-3

**Published:** 2020-01-08

**Authors:** Yuan Gao, Yanlin Niu, Wanwan Sun, Keke Liu, Xiaobo Liu, Ning Zhao, Yujuan Yue, Haixia Wu, Fengxia Meng, Jun Wang, Xueshuang Wang, Qiyong Liu

**Affiliations:** 1 State Key Laboratory of Infectious Disease Prevention and Control, Collaborative Innovation Center for Diagnosis and Treatment of Infectious Diseases, National Institute for Communicable Disease Control and Prevention, Chinese Center for Disease Control and Prevention, Beijing, China; 2 Beijing Center for Diseases Prevention and Control, Beijing, China

**Keywords:** Typhus group rickettsiosis, Climate, Distributed lag non-linear models, China

## Abstract

**Background:**

Typhus group rickettsiosis (TGR), which is a neglected vector-borne infectious disease, including epidemic typhus and endemic typhus. We explored the lag effects and nonlinear association between meteorological factors and TGR incidence in Xishuangbanna Dai autonomous prefecture from 2005 to 2017, China.

**Methods:**

A Poisson regression with a distributed lag nonlinear model (DLNM) was utilized to analyze TGR cases data and the contemporaneous meteorological data.

**Results:**

A J-shaped nonlinear association between weekly mean temperature and TGR incidence was found. The cumulative exposure to weekly mean temperature indicated that the RR increased with the increment of temperature. Taking the median value as the reference, lower temperatures could decrease the risk of TGR incidence, while higher temperatures could increase the risk of TGR incidence and last for 21 weeks. We also found a reversed U-shaped nonlinear association between weekly mean precipitation and TGR incidence. Precipitation between 5 mm and 13 mm could increase the risk of TGR incidence. Taking the median value as the reference, no precipitation and lower precipitation could decrease the risk of TGR incidence, while higher precipitation could increase the risk of TGR incidence and last for 18 weeks.

**Conclusions:**

The prevention and control measures of TGR should be implemented according to climatic conditions by the local government and health departments in order to improve the efficiency.

## Background

Typhus group rickettsiosis, which is a neglected vector-borne infectious disease, including epidemic typhus and endemic typhus, is caused by *Rickettsia prowazekii* and *Rickettsia typhi,* respectively [1]. Epidemic typhus or louse-borne typhus, is usually transmitted through the human body louse [2]. Endemic typhus or murine or flea-borne typhus, is usually transmitted by fleas. The main transmission cycle is rat-fleas, and other transmission cycles, including fleas from opossums, dogs, and cats, are reported worldwide [3–5]. Rare epidemic typhus outbreaks have been reported during past several decades. However, murine typhus is widely distributed around the world. The incubation period for murine typhus is 8 to 12 days. Murine typhus could be a self-limiting clinical presentation with mild symptoms, such as fever, headache, exanthema, muscle pain, joint pain and vomiting [6]. However, sometimes murine typhus may cause severe complications if the diagnosis and treatment are not timely [7]. In recent years, many regions have reported murine typhus cases with severe complications, such as pneumonia, pancreatitis, and septic shock [8–10]. There are only a few regions still monitor murine typhus, such as Texas, Hawaii, California and Taiwan [7, 11]. Moreover, murine typhus poses a threat to the health of travelers, and travel destinations primarily locate in Southeast Asia, Africa and America [12].

TGR has been reported as a Class C notifiable communicable disease which should be reported within 24 h after being diagnosed in China. It was reported that the main type of TGR in China was endemic typhus, and the main vector was *Xenopsylla cheopis* [13–15]. TGR is widely distributed in mainland China with a total of 29,211 TGR cases located in 29 provinces and 795 counties from 2005 to 2017. Among the cases, there were 9129 cases in Xishuangbanna Dai autonomous prefecture of Yunnan province, accounting for 31.25% of all cases in China. The annual incidence here ranged from 105.87 in 2011 to 10.67 in 2017 per 100,000 individuals, greater more than the average incidence in China (0.16/100,000). According to the precious study, under suitable climate conditions, *Xenopsylla cheopis* had a survival period of 377 days, and an average life span of 172.4 days. The optimum growth temperature was 23 °C, and at this temperature, the breeding cycle was generally 20 to 44 days [16]. The possible routes of transmission are flea bites, contamination of excoriated skin, and inhalation of contaminated aerosols [5]. According to our previous study, we found that TGR was sensitive to the climate, and most cases occurred from May to October. Meteorological factors may affect TGR incidence both directly and indirectly by affecting vector ecology, vector–human interactions, and bacterial reproduction. Li et al. used Pearson correlation analysis to analyze the correlation between meteorological factors and TGR incidence from 2005 to 2013 in Baoshan city, Yunnan province. They concluded that temperature and precipitation were closely related to TGR incidence [17]. However, the study neglected the nonlinear relationship and lag effects between meteorological factors and TGR incidence.

Distributed lag non-linear models (DLNMs) represent a modeling framework to flexibly describe associations showing potentially nonlinear and delayed effects in time series data. This methodology rests on the definition of a crossbasis, a bi-dimensional functional space expressed by the combination of two sets of basis functions, which specify the relationships in the dimensions of predictor and lags, respectively [18]. DLNMs have been widely used to analyze air pollution on years of life lost, mortality, hospital admissions and so on [19–21]. Furthermore, in recent years, DLNMs have been applied to study association between meteorological factors and communicable diseases, for example, dengue, hand, foot and mouth disease, severe fever with thrombocytopenia syndrome (SFTS) [22–24]. In detail, Sun et al. found a reversed U-shaped nonlinear relationship between ambient temperature and SFTS [24]. However, no study has been conducted to study the association between meteorological factors and TGR incidence. Therefore, we used DLNM to explore the temporal lag association between meteorological factors and TGR incidence. Results can be used as an early warning for public health authorities, and have a better understanding of TGR ecology.

## Methods

### Study area

**Fig. 1:**
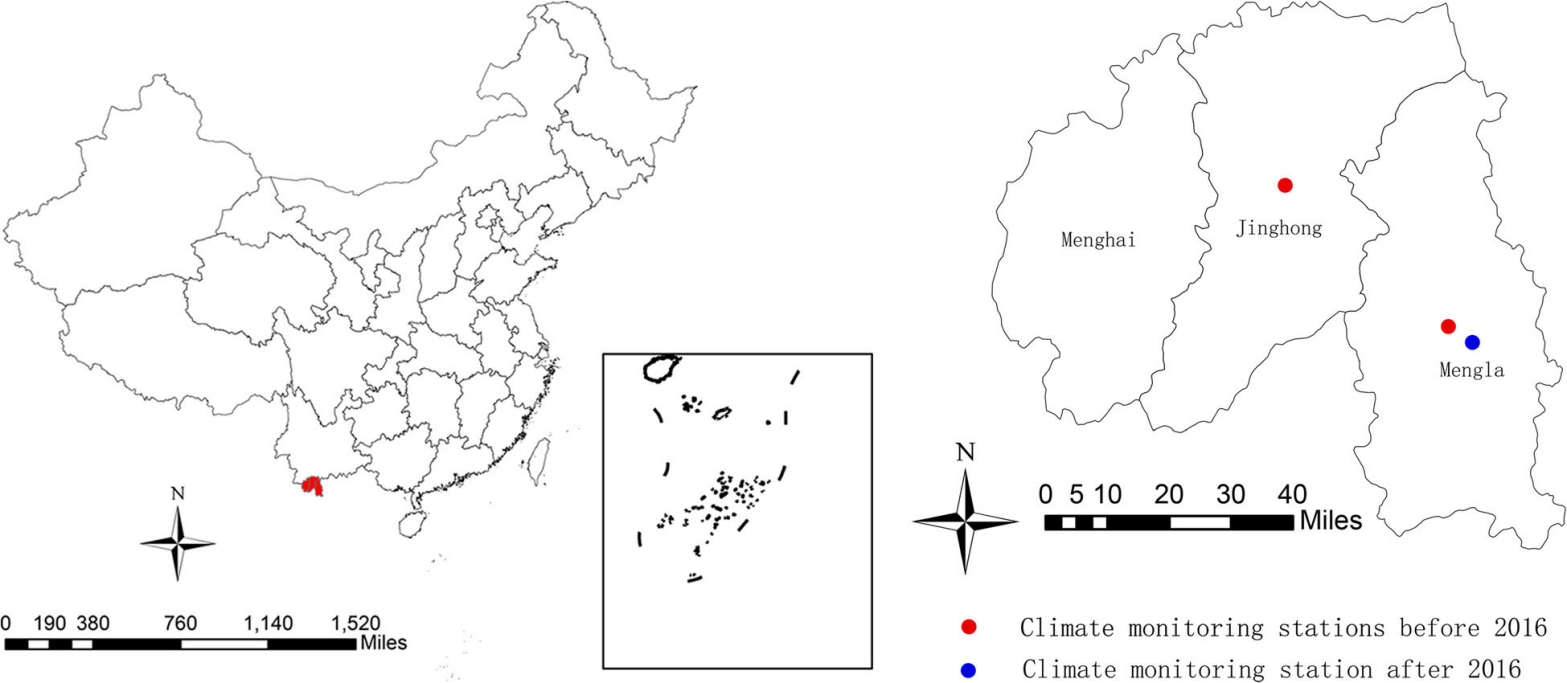
Location of Xishuangbanna Dai autonomous prefecture of Yunnan province in China and the climate monitoring stations

In our previous study, we identified the primary cluster of TGR where had the highest TGR incidence rate using SaTScan software (version 9.4.4). The primary cluster was located in Xishuangbanna Dai autonomous prefecture, including three counties, Jinghong city, Menghai county and Mengla county of Yunnan province. Xishuangbanna Dai autonomous prefecture (latitude 21°10′-22°40′N, longitude 99°55′-101°50′E) is situated in the southwestern part of China (Fig. 1), bordering Burma to the southwest and Laos to the southeast, with a population of about 1,130,000 in 2010. The climate here is mild, warm and humid all year round with an annual average temperature from 18.6 to 22.9 °C, and an annual precipitation from 1347.4 to 1916.8 mm [25]. Therefore, it is rich in animals and plants and has developed tourism. According to the previous study, the type of TGR here was dominated by endemic typhus [13]. A total of 9,129 TGR cases were reported from 2005 to 2017.

### Data collection

Daily data of TGR cases from 2005 to 2017 were obtained from the China Information System for Diseases Control and Prevention. Epidemic typhus or endemic typhus was not divided in the system. TGR was diagnosed according to the diagnostic criteria principles of management for TGR (WS 215–2001, before 2008) or diagnostic criteria for TGR (WS 215–2008, after 2008), issued by the Ministry of Health of the People’s Republic of China [26]. Clinical diagnosed cases and confirmed cases were adopted in our study. Daily meteorological data including mean temperature (Tmean), maximum temperature (Tmax), minimum temperature (Tmin), mean relative humidity (Hmean) and mean cumulative precipitation of the study area were collected from the China Meteorological Data Sharing Service System (http://data.cma.cn/). There were two climate monitoring stations, located in Mengla county and Jinghong city before 2016. And after 2016, there was only one climate monitoring station located in Mengla county (Fig. 1).

### Data analysis

Descriptive analysis was utilized to demonstrate the characteristics of TGR cases and meteorological factors. The correlation between TGR cases and meteorological factors was decided by the Spearman’s rank correlation coefficient with a significance of *P* < 0.05.

Considering the nonlinear and lag influence of meteorological factors on the TGR incidence, DLNM in the R package was applied to graphically demonstrate the three-dimensional (3D) meteorological factors–TGR–lag effects. DLNM is based on the combination of the generalized additive model and the distributed lag linear model. The core idea is to add a lag dimension to the exposure-response relationship through the cross-basis function which captures the exposure-lag-response dependency simultaneously. We had evaluated the interaction of each variable in one generalized additive model, the results showed that TGR cases were best predicted as positive smooth functions in the weekly mean temperature and mean cumulative precipitation. Considering that there were significant correlations among Tmean, Tmax and Tmin (Additional file 2: Table S1), so we introduced them into the model separately. Finally, we built two separate models to explore individual lag effects of different factors, including weekly mean temperature and cumulative precipitation, using DLNM models.

A natural cubic spline DLNM was applied to model the nonlinear relationship. In order to avoid over-dispersion of the time series data, a quasi-Poisson distribution was adopted as the connection function of the DLNM. The maximum degree of freedom was set to 4 in the model selection. A quasi-Akaike Information Criterion (QAIC) was utilized to select the degrees of freedom for meteorological factors and lag. Lower QAIC was preferred.

The basic model structure applied in our study was given as follows:

$$ \mathrm{Log}\left[\mathrm{E}\left({\mathrm{Y}}_{\mathrm{t}}\right)\right]=\upalpha +\sum \mathrm{ns}\left({\mathrm{weather}}_{\mathrm{t}},\mathrm{df}=4\right)+\mathrm{as}.\mathrm{factor}\ \left(\mathrm{week}\right)+\mathrm{as}.\mathrm{factor}\ \left(\mathrm{year}\right)+\mathrm{cb}\left(\mathrm{t},\mathrm{l}\right) $$

Where *t* is the week of the observation; *Y*_*t*_ is the observed weekly number of TGR cases in week *t*; *α* is the intercept; *ns(.)* is a cubic spline function; the long-term and seasonal trends are adjusted using a factor of year and week ordinal; weather variables are adjusted using a ns with a maximum of 4 df. *cb(.)* is a matrix produced by DLNM to model nonlinear and distributed lag effects of the weekly mean temperature and precipitation, respectively. Specifically, we use a cubic B-spline with four equally spaced knots in the log scale. To date, there is no reference for selecting the lag in TGR. So the maximum of lag was selected by the graphical effects and the results of cross correlation function which included lagged correlation (Additional file 1). The maximum lag of weekly mean temperature was 21 weeks, and the maximum lag of weekly mean precipitation was 18 weeks. The median values were applied as the reference to estimate the relative risks, respectively. The relative risks of TGR incidence were estimated by different meteorological factor structures (5_th_,25_th_, 75_th_, 95_th_ percentile) relative to the reference value.

The final model was a natural cubic spline of weekly meteorological factors with three degrees of freedom for weekly mean temperature, four degrees of freedom for weekly mean relative humidity and four degrees of freedom for weekly precipitation, respectively.

The “DLNM” and “spline” packages in the R software were used to create the DLNM model. All data analysis were completed in R software (version 3.5.1).

## Results

A total of 9,129 TGR cases were collected from 2005 to 2017 in the study area, accounting to 31.25% of the whole cases in China. The summary of weekly TGR cases, Tmean, Tmax, Tmin, Hmean and precipitation was shown in Table 1.

**Table 1:** Summary of weekly typhus group rickettsiosis cases and meteorological variables in Xishuangbanna Dai autonomous prefecture of Yunnan province in China, 2005–2017

	Minimum	First quartile	Median	Mean	Third quartile	Maximum
Number of typhus group rickettsiosis cases	0.00	5.00	12.00	13.44	20.00	55.00
Mean temperature (°C)	11.15	19.45	23.60	22.52	25.39	27.99
Maximum temperature (°C)	16.48	28.13	30.76	30.03	32.05	37.26
Minimum temperature (°C)	7.20	14.53	19.42	18.38	22.39	23.98
Mean relative humidity (%)	56.79	74.39	80.36	78.81	83.89	94.00
Precipitation (mm)	0.00	0.08	2.09	3.82	6.13	26.71

**Fig. 2:**
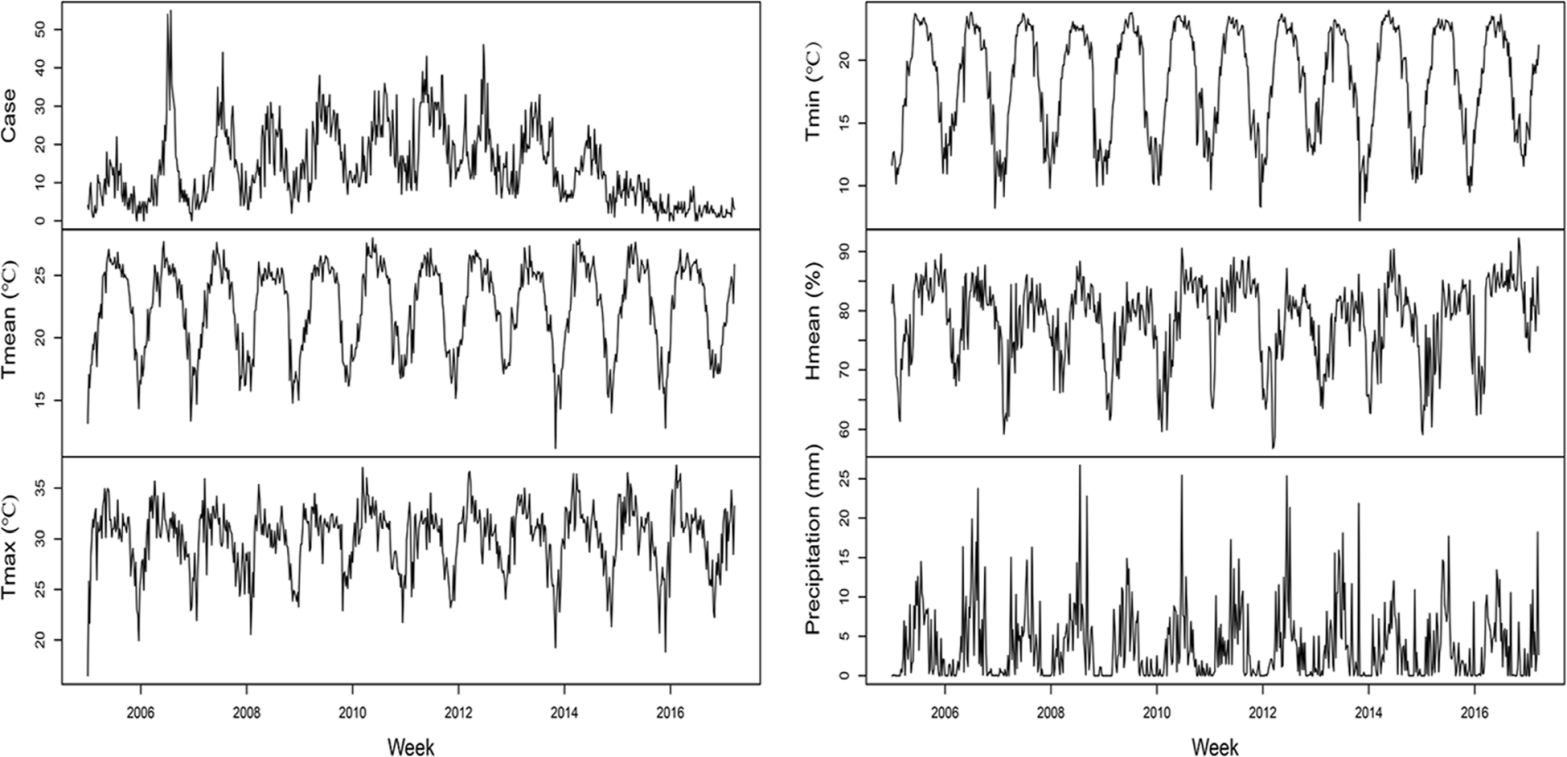
Weekly time series distributions of typhus group rickettsiosis cases and meteorological factors from 2005 to 2017 in Xishuangbanna Dai autonomous prefecture of Yunnan province in China

The time series distributions of weekly TGR cases and meteorological factors were shown in Fig. 2. The results indicated that TGR cases had obvious seasonal distribution characteristics with higher incidence in summer and autumn. There were different seasonal patterns of different meteorological factors.

**Fig. 3:**
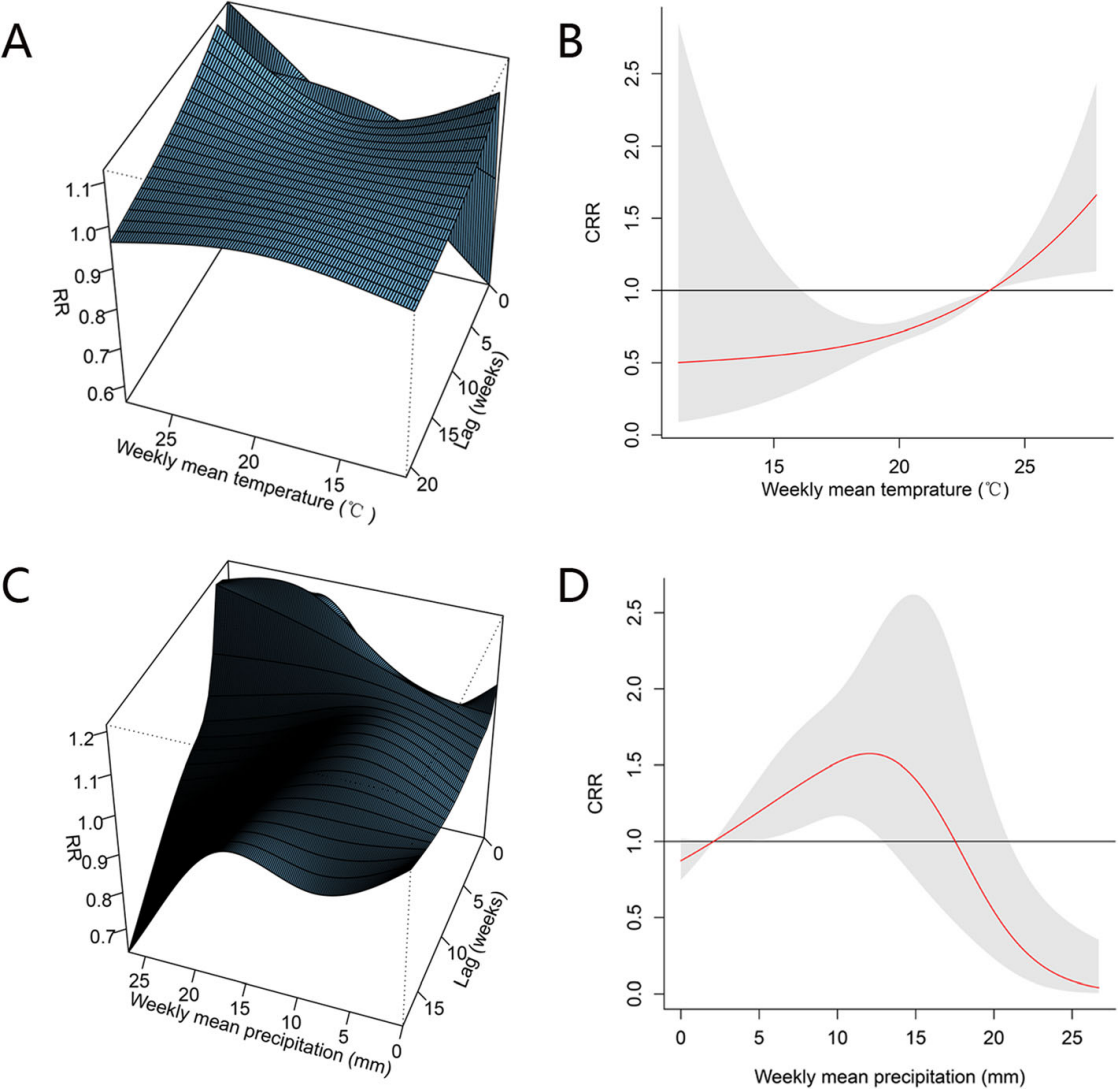
Three-dimensional plots and overall relative risk plots of the relationship between weekly mean temperature and precipitation and typhus group rickettsiosis in Xishuangbanna Dai autonomous prefecture. **a**. Three-dimensional plot of the relationship between weekly mean temperature and typhus group rickettsiosis over 21 lag weeks. **b**. Overall relative risks of weekly mean temperature for typhus group rickettsiosis cases over 21 lag weeks. **c**. Three-dimensional plot of the relationship between weekly mean precipitation and typhus group rickettsiosis over 18 lag weeks. **d**. Overall relative risks of weekly mean precipitation for typhus group rickettsiosis cases over 18 lag weeks

The 3D weekly meteorological factors-TGR relationship along with lag weeks and overall relative risk plots of the relationship between weekly mean temperature, precipitation and TGR were shown in Fig. 3. Figure 3 a shows the 3D plot of weekly mean temperature and TGR incidence among 21 lag weeks. The estimate effects of mean temperature on TGR incidence were nonlinear, with a peak RR at the highest mean temperature by lag 0. A J-shaped nonlinear association between weekly mean temperature and TGR incidence was found. The cumulative exposure to weekly mean temperature indicated that the RR increased with the increment of temperature. Temperature greater than 24 °C increased the risk of TGR incidence (Fig. 3 b). Figure 3 c shows the 3D plot of weekly mean precipitation and TGR incidence among 18 lag weeks. The highest risk was observed at lag 0 when the precipitation was between 15 mm and 20 mm. A reversed U-shaped nonlinear association between weekly mean precipitation and TGR incidence was found. Precipitation between 5 mm and 13 mm could increase the risk of TGR incidence with a peak relative risk at roughly 13 mm (Fig. 3 d).

**Fig. 4:**
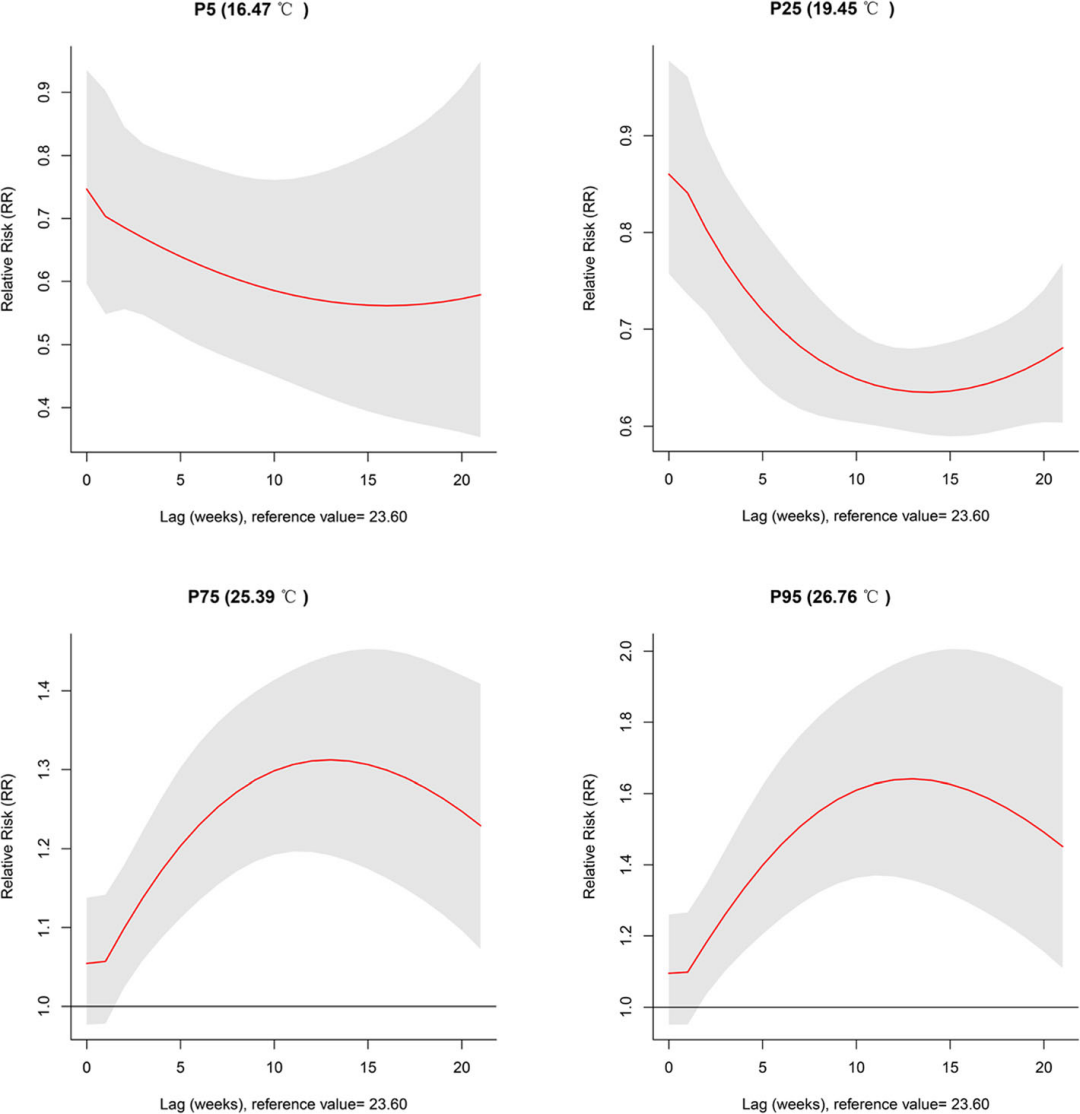
Plots of relative risk (RR) by lag at specific temperatures

The relative risk of mean temperature was illustrated by lag at specific temperatures, 5_th_ (16.47 °C), 25_th_ (19.45 °C), 75_th_ (25.39 °C), and 95_th_ (26.76 °C) percentiles (Fig. 4). The results showed that taking the median as a reference value, lower temperatures (5_th_ and 25_th_) could decrease the risk of TGR incidence, while higher temperatures (75_th_ and 95_th_) could increase the risk of TGR incidence from lag week 2 and the lag effects lasted for 21 weeks with the highest risk at roughly about lag 10 to 15 weeks (Additional file 3: Table S2).

**Fig. 5:**
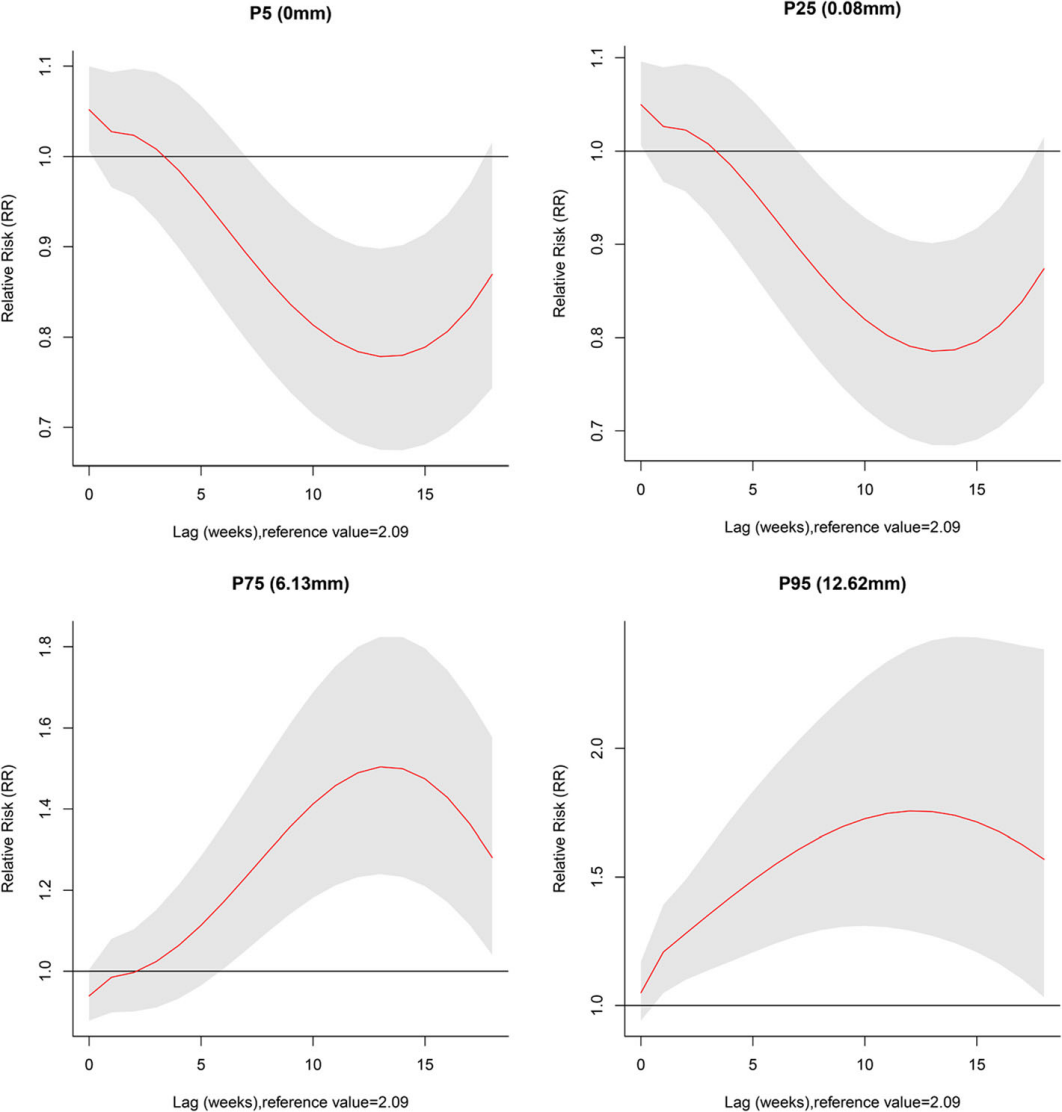
Plots of relative risk (RR) by lag at specific precipitation

Figure 5 shows the RR by lag at specific precipitation, 5_th_ (0 mm), 25_th_ (0.08 mm), 75_th_ (6.13 mm), and 95_th_ (12.62 mm) percentiles of weekly mean precipitation. The results showed that taking the median as a reference value, no precipitation and lower precipitation (5_th_ and 25_th_) could decrease the risk of TGR incidence, while higher precipitation (75_th_ and 95_th_) could increase the risk of TGR incidence from lag week 6 and lag week 2, respectively. They both had long-term effects with the lag effects lasted for 18 weeks. The highest risk was found at lag 12 weeks (Additional file 3: Table S2)

## Discussion

Xishuangbanna Dai autonomous prefecture is on the northern edge of the tropics, belonging to the tropical rainforest climate. There are abundant precipitation and sunshine here, with a sufficient annual precipitation between 1136 mm and 1513 mm and an annual mean temperature between 18.9 °C and 22.6 °C. In our study, we explored the graphical and quantitative lag associations between temperature, precipitation and TGR incidence between 2005 and 2017 using DLNM.

There were some previous studies suggesting that TGR incidence was related to meteorological factors. Kuo et al. found murine typhus was negative related to precipitation and temperature after controlling the distance of operating international seaports using a Bayesian negative binomial conditionally autoregressive model [11]. Li et al. reported that TGR incidence was positive associated with precipitation and temperature using Pearson correlation analysis [17]. There were studies reporting that there was a correlation between murine typhus and hot season [27, 28]. In our study, we found there were lag effects of temperature, and precipitation on TGR incidence. The results showed that taking the median as a reference value, lower temperatures (5_th_ and 25_th_) could decrease the risk of TGR incidence, while higher temperatures (75_th_ and 95_th_) could increase the risk of TGR incidence from lag week 2 and the lag effects lasted for 21 weeks with the highest risk at roughly about lag 10 to 15 weeks in Xishuangbanna Dai autonomous prefecture. The effects at high temperature ranges were persistent over longer lag periods. A J-shaped nonlinear overall effect was found between weekly mean temperature and TGR incidence. This results consist with the habits of *Xenopsylla cheopis* fleas. In the laboratory settings, the suitable temperature for *Xenopsylla cheopis* breeding was higher than around 24 °C [29]. This results fully suggest that the type of TGR in Xishuangbanna is dominated by murine typhus. Temperature may affect many aspects of the TGR epidemiologic directly and indirectly, for example, vector growth, bacterial reproduction, host activities, human activities and their interactions. In addition, appropriate temperature may influence human activities. In detail, humans are more likely to conduct outdoor activities, so they have more exposure opportunities.

We also found a reversed U-shaped nonlinear association between precipitation and TGR incidence. This results indicate that there is a threshold precipitation for TGR transmission. Precipitation between 5 mm and 13 mm could increase the risk of TGR incidence with a peak relative risk at roughly 13 mm, taking the median value as the reference. Appropriate precipitation is conducive to the growth and reproduction of rats and fleas. However, a direct effect occurs when high intensity precipitation causes flooding of rodent burrows [30]. As observed in this study, the risk of TGR cases vanished when precipitation exceeded 13 mm.

In fact, the cycle of vector-borne disease system and climate is very complex. The densities, life cycle, dynamics and geographical distributions of vectors and hosts are all individually influenced by climate variables [30]. Warm-moist climate seems more likely to form high flea indices [31]. We found that there were lagged effects of temperature and precipitation on TGR. Weekly mean temperatures over 23 °C, and precipitation between 5 mm and 13 mm, may lead to higher flea densities and further breeding in subsequent generations. It means that targeted interventions, such as health education, rodents and fleas control, doctors training, should be implemented in the study area. Further studies should be implemented to explore the underlying mechanism.

There are several limitations in our study. First, the results might not be appropriate for other regions with different climate zones. Second, our study explored the exposure-response-lag relationship on a weekly scale which might affect the preciseness of exposure. Third, TGR occurrence is related to the complex interactions between humans, vectors, hosts, and the bacteria. Other risk factors, such as vector or host intensity, environmental hygiene and socioeconomic conditions, were not taken into account when modelling the climate-TGR associations. Moreover, it is worthwhile analyzing the influence of EI Niño-Southern Oscillation (ENSO) using Sea Surface Temperatures (SST) or Oceanic Nino Index (ONI) as explanatory variable in the next step.

## Conclusions

In conclusion, our study broadens new knowledge of association between TGR incidence and meteorological factors. We explored the nonlinear and lag associations between weekly mean temperature, precipitation and TGR incidence on a weekly scale systematically. However, more investigations of underlying mechanism of different lag patterns should be explored. The prevention and control measures of TGR should be implemented according to climatic conditions by the local government and health departments in order to improve the efficiency.

## Supplementary information

**Additional file 1.** Supplementary material 1: The PACF plots and residual deviation plots of two individual models. Supplementary material 2: The results of cross correlation function

**Additional file 2: Table S1.** Correlation analysis of TGR cases and meteorological factors using Spearman’s correlation test

**Additional file 3.** The relative risks of different factors at different lag weeks

## Data Availability

The datasets used and analyzed during the current study are available from the corresponding author on reasonable request.

## Abbreviations

3D: Three-Dimensional
DLNM: Distributed Lag Non-linear Model
ENSO: EI Niño-Southern Oscillation
Hmean: Mean relative humidity
ONI: Oceanic Nino Index
SFTS: Severe Fever with Thrombocytopenia Syndrome
SST: Sea Surface Temperatures
TGR: Typhus Group Rickettsiosis
Tmax: Maximum temperature
Tmean: Mean temperature
Tmin: Minimum temperature
