# Supplementary material for: Climate factors driven typhus group rickettsiosis incidence dynamics in Xishuangbanna Dai autonomous prefecture of Yunnan province in China, 2005–2017
Source: Environ Health. 2020 Jan 8;19:3. doi: 10.1186/s12940-019-0558-3 (PMC6951009; doi:10.1186/s12940-019-0558-3)
Supplement: Supplementary file 1 — Additional file 1. Supplementary material 1: The PACF plots and residual deviation plots of two individual models. Supplementary material 2: The results of cross correlation function [file 12940_2019_558_MOESM1_ESM.docx]

Supplementary material

Supplementary material 1: The PACF plots and residual deviation plots of two individual models.


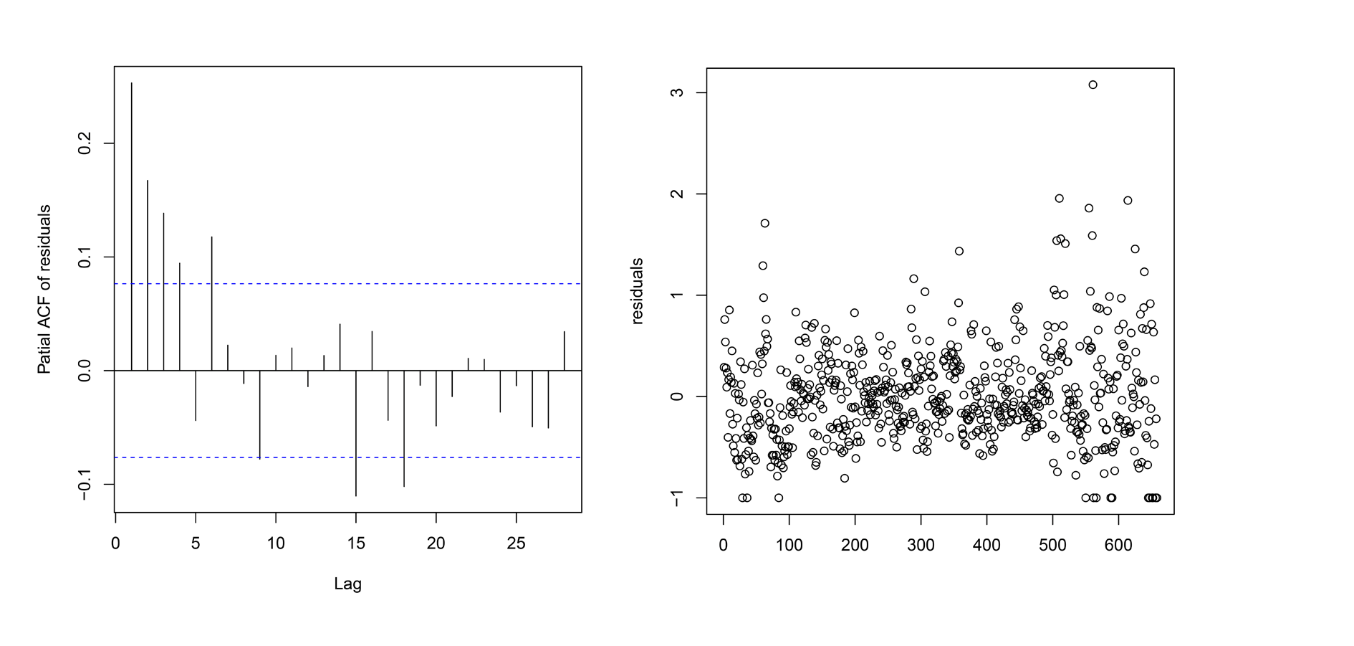
Model 1:

Model 2:


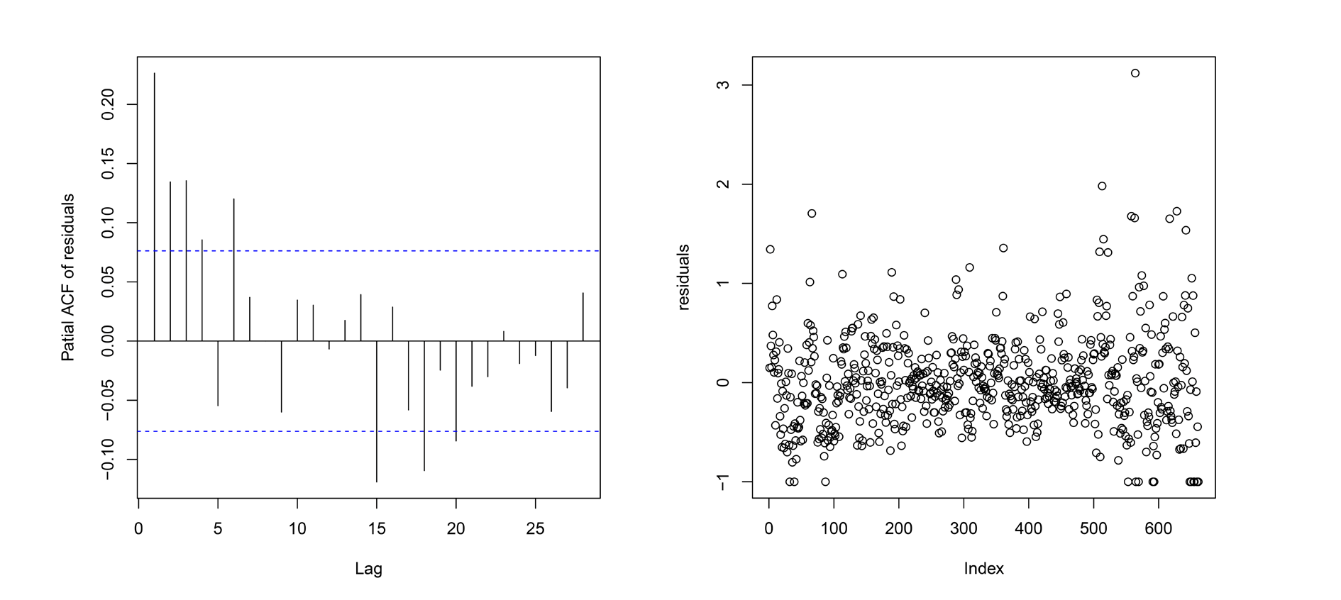


Supplementary material 2: The results of cross correlation function.


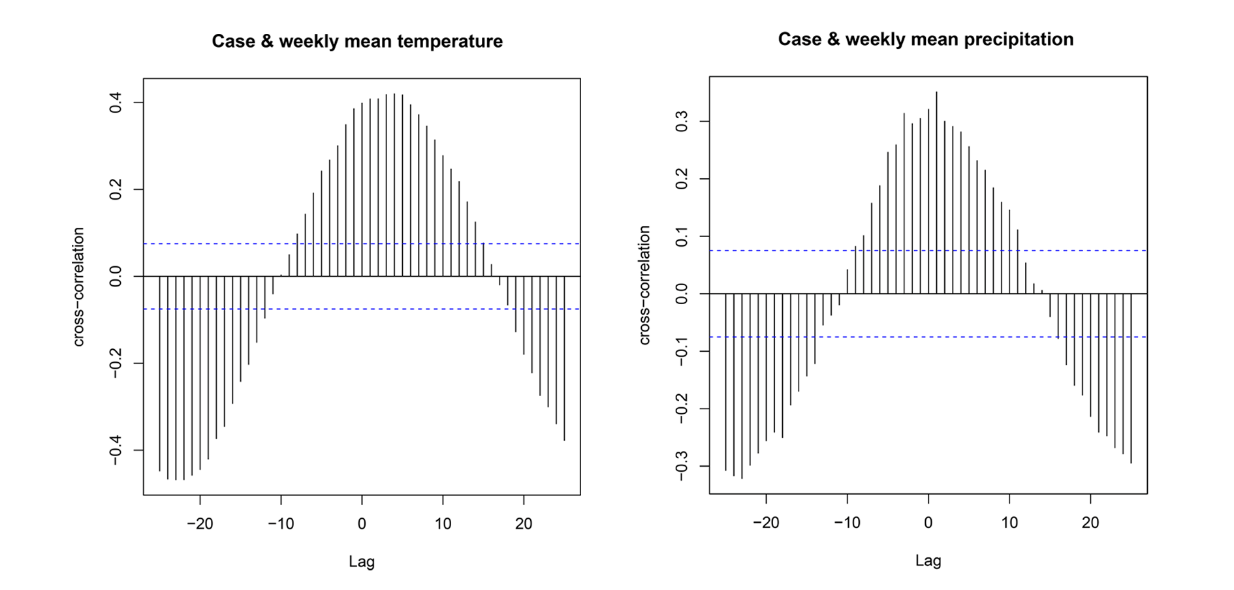


Model 1

Model 2
